# WHO 2016 Definition of Chronic Myeloid Leukemia and Tyrosine Kinase Inhibitors

**DOI:** 10.4274/tjh.galenos.2019.2019.0241

**Published:** 2020-02-20

**Authors:** İbrahim C. Haznedaroğlu, Işınsu Kuzu, Osman İlhan

**Affiliations:** 1 Hacettepe University Faculty of Medicine, Department of Hematology, Ankara, Turkey; 2 Ankara University Faculty of Medicine, Department of Pathology, Ankara, Turkey; 3 Ankara University Faculty of Medicine, Therapeutic Apheresis Unit, Department of Hematology, Ankara, Turkey

**Keywords:** Chronic myeloid leukemia, CML, Tyrosine kinase inhibitor, TKI

## Abstract

Philadelphia (Ph*)/BCR-ABL1-positive chronic myeloid leukemia (CML) is considered as a chronic life-long disease, which could be manageable with tyrosine kinase inhibitor (TKI) drugs. The aim of TKI drug treatment is to provide age- and sex-matched duration of life in a given patient with CML. Personalized CML treatment with TKI drugs is the key strategy. Individual treatment approach includes the harmonization of CML disease characteristics, clinical experience, and best available clinical evidence. Specific CML disease characteristics in a given patient include; CML disease risk, comorbidities, molecular profile, compliance, lifestyle, and drug off-target risk profile. CML research evidence includes; randomized clinical trials indicating the data on the efficacy, safety, tolerability, toxicity, possible long-term adverse events, and pharmacoeconomy of TKIs. Clinical and physician experience includes TKI availability, TKI reimbursability, drug experience, adherence, and BCR-ABL1 monitorization facilities. The key decision of choosing a TKI of choosing TKIs for CML should be made via the consideration of these variables. The aim of this paper is to outline the latest 2016 World Health Organization definition of CML and its proper management with TKI-class drugs.

## Abstract (tr)

Philadelphia (Ph*)/BCR-ABL1 (+) kronik myeloid lösemi (KML), tirozin kinaz inhibitörleri (TKİ) grubundan ilaçlarla yaşam boyu yönetilebilecek kronik bir hastalıktır. TKİ ilaç tedavisinin hedefi, herhangi bir KML hastasında aynı yaş ve cinsiyette sağlıklı bireylerde beklenen yaşam süresi idamesini sağlamak olmalıdır. KML tedavisinde bireyselleştirilmiş TKİ ilaç kullanımı anahtar stratejidir. Bireysel tedavi yaklaşımı; KML hastalık özelliklerini, klinik deneyimi ve mevcut en iyi kanıtı uygunca birleştirme esaslıdır. Herhangi bir KML hastasında özgül hastalık özellikleri; KML hastalık riski, komorbiditeler, moleküler profil, hasta uyumu, yaşam tarzı, ve ilaç temelli yan etki profilleridir. KML’de kritik araştırma kanıtları; TKİ etkinlik, güvenilirlik, tolerabilite, toksisite, uzun-dönem ilaç yan etkileri ve farmakoekonomi parametreleri için karar verdirici nitelikte olan randomize klinik çalışmalardır. Klinik ve hekim deneyimi; TKİ mevcudiyeti, TKİ geriödenebilirliği, ilaç deneyimi, ilaca uyum ve izleyen klinikte BCR-ABL1 izlem olanakları olarak özetlenebilir. KML seyrinde ana kritik TKİ kararına esas olarak sayılan bu değişkenlerin dikkate alınması sonrasında ulaşılır. Bu makalenin amacı, KML tanımlamasında en son kullanılan Dünya Sağlık Örgütü-2016 kriterleri eşliğinde TKİ grubu ilaçlar ile uygun KML yönetimi ilkelerini tartışmaktır.

## Introduction

**Figure 1:**
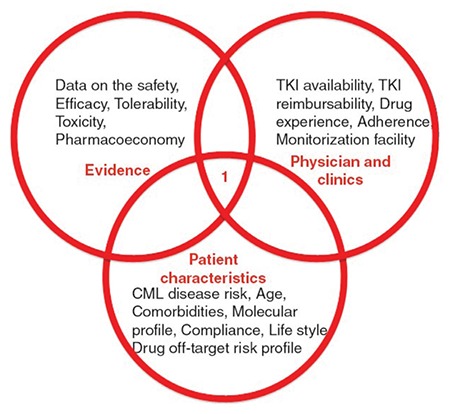
The harmonization of individual disease characteristics, the experience of physician/clinical facilities, and best clinical evidence is essential for clinical decision-making in chronic myeloid leukemia (CML). CML: Chronic myeloid leukemia, TKI: Tyrosine kinase inhibitor.

Philadelphia (Ph*)/BCR-ABL1-positive chronic myeloid leukemia (CML) is a chronic neoplastic disease, which can be functionally cured via the administration of tyrosine kinase inhibitor (TKI) drugs [1]. The overall aim of TKI therapy in CML is to provide normal life duration and quality to the patient. The harmonization of CML disease characteristics, physician/clinic facilities, and best clinical evidence is vital to reach this ultimate aim [2,3]. The disease characteristics of a given patient include CML disease risk, comorbidities, molecular profile, compliance, lifestyle, and drug off-target risk profile. CML research evidence includes randomized clinical trials indicating data on the safety, efficacy, tolerability, toxicity, possible long-term adverse events, and pharmacoeconomy of TKIs. Clinical experience involves TKI availability, TKI reimbursability, drug experience, adherence, and monitorization facilities. The critical decision regarding TKIs for CML should be done via the optimization of those variables for every single CML patient (Figure 1) [3]. The aim of this paper is to outline the proper TKI treatment for the management of CML, as described in the 2016 World Health Organization (WHO) classification [3].

## 2016 WHO Definition of Chronic Myeloid Leukemia

The essential clinicopathological characteristics of Ph*(+) CML in the 2016 WHO classification are defined as follows [4];

### Chronic Phase CML

This is a myeloproliferative neoplasm characterized by the chromosomal translocation t(9;22) (q34.1;q11.2), resulting in the BCR-ABL1 fusion gene and formation of the Philadelphia chromosome (Ph*), which causes an increase in blood granulocytes and bone marrow myeloid precursors as the major proliferative component. Cryptic and variant forms of the Philadelphia chromosome as well as additional cytogenetic abnormalities may complicate the disease pathobiology. Therefore, interphase fluorescence in situ hybridization (FISH), chromosome banding analysis, and PCR should be integrated for the diagnosis and follow-up of CML [5,6].

The disease is described in three main clinical phases, which were significantly prognostic before the TKI treatment era. The chronic phase is the initial phase. Disease progression is then described in two phases as the accelerated phase (AP) and blastic phase (BP). AP disease is characterized by 10%-19% blasts in the bone marrow or peripheral blood. The criterion for transformed BP is more than 20% blasts either in the blood or in the bone marrow, or at extramedullary sites [4].

**Figure 2:**
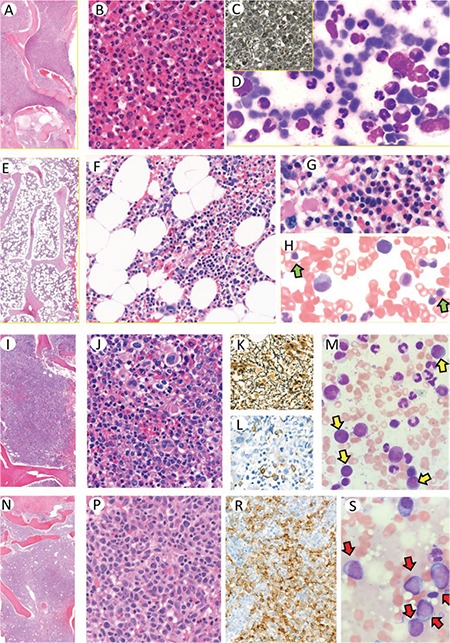
Bone marrow biopsy in chronic phase (CP) CML is usually hypercellular with 100% cellularity **(A)**. The bone marrow cells are almost all composed of mature granulocytes and their precursors **(B)**. Reticulin could be seen, especially in the cases with increased megakaryocytes, but usually does not increase **(C)**. Bone marrow aspirate is hypercellular, composed of maturing granulocytic precursors with striking decrease in other precursors **(D)**. Cellularity decreases in the bone marrow of responders to TKI treatment **(E, F)**. The islands of erythroid precursors and megakaryocytes as well as the granulocytic series reflect the normal composition **(G)**. Aspirate smears can also reflect the normal cellular composition with erythroid precursors (**H**; green arrows). Accelerated phase (AP) CML is characterized by increased blasts of <10%-19% and/or megakaryocytes **(I, J)**. Increase in megakaryocyte population promotes reticulin fibrosis **(K)**. Immunohistochemistry is helpful, especially for demonstrating blasts by CD34 staining **(L)**. Blasts on bone marrow aspirates are scattered between myeloid precursors (**M**; yellow arrows). The blasts are the dominant cellular component in the bone marrow of blastic phase (BP) CML **(N, P)**. Presence of strikingly increased blasts could be demonstrated by CD34 immunohistochemistry **(R)**. On bone marrow aspirate smears, blastic cells are also dominant (**S**; red arrows).

Typical peripheral blood findings in CP-CML are characterized by increased neutrophils with various early-stage granulocytic precursors. The diagnosis needs to be proven by demonstrating the molecular abnormality of BCR-ABL1 fusion. Typical bone marrow (BM) histopathology is demonstrated in Figures 2A-2D.

The presence of t(9;22) (q34.1;q11.2) or BCR-ABL1 abnormality could be demonstrated by karyotype analysis, FISH, or PCR-based methods. The most reliable and sensitive method is real-time PCR. This method is important and should be preferred especially for routine monitoring for the evaluation of the response to TKI treatment [7].

Complete responders to TKI treatment are defined by <10x109/L blood cell count and <450x109/L platelet count without any immature granulocytes in differentiation and nonpalpable spleen [4]. The bone marrow features and cellular compositions are normal with the appearance of erythrocytic precursors. Such a case is demonstrated in Figures 2E-2H.

### Accelerated Phase CML

The typical BM histopathology for AP was described before the TKI treatment era. Following the start of the TKI era, the criteria were modified considering the therapy. Cases responding to TKI treatment are characterized by normalization of the cellular composition of the bone marrow as demonstrated in Figures 2I-2M. Abnormal megakaryocytes associated with marked reticulin or collagen fibrosis in accordance with typical AP-CML could be present (Figure 2K). The AP criteria are listed below [4].

• The presence of t(9;22)(q34.1;q11.2) or BCR-ABL1 (via molecular biology or karyotype analyses) together with genomic cytogenetic evolution and/or TKI resistance.

• Genomic evolution may include second Ph*, trisomy 8, isochromosome 17q, trisomy 19, complex karyotype, or 3q26.2 abnormalities.

• Persistent or increasing abnormal blood counts despite TKI treatment (leukocytosis (>10x109/L), thrombocytosis (>1000x109/L), or thrombocytopenia (<100x109/L) unrelated to therapy, 20% or more basophils, 10%-19% blasts)

• Persistent or increasing splenomegaly.

• Occurrence of clinically significant driver mutations in BCR-ABL1 during TKI therapy (particularly T315I).

• Additional clonal chromosomal abnormalities such as trisomy 8, isochromosome 17q, trisomy 19, or any new entity of complex karyotype and 3q26.2 abnormalities or any new chromosomal abnormality in BCR-ABL fusion-positive cells occurring during TKI treatment are the accepted criteria

There are also provisional response criteria to TKI treatment as described in the 2016 WHO classification. These are: 1- Failure to achieve complete response to the TKI treatment or hematological resistance; 2- Any hematological, cytogenetic, or molecular indications of resistance to TKI treatment; 3- Occurrence of two or more mutations in the BCR-ABL fusion gene during TKI therapy [4].

### Blastic Phase CML

Typical BM histopathology is presented in Figures 2N-2S with increased blastic infiltration in accordance with the typical BP-CML clinical presentation. The relevant criteria follow [4].

• The presence of t(9;22)(q34.1;q11.2) or BCR-ABL1 (via molecular biology or karyotype analyses) together with genomic cytogenetic evolution and/or TKI resistance.

• Genomic evolution may include second Ph* chromosome, trisomy 8, isochromosome 17q, trisomy 19, complex karyotype, or 3q26.2 abnormalities.

• The presence of at least 20% blasts in the peripheral blood and/or BM or the presence of extramedullary blastic infiltration in any organ or tissue

• Persistent or increasing splenomegaly.

## Frontline Strategies for CML Patients

TKI drug treatment should be initiated as soon as possible in patients newly diagnosed with CML. The aim of chronic TKI therapy in CML is the restoration of normal hematopoiesis instead of the neoplastic BCR-ABL1-induced myeloid neoplastic proliferation and the prevention of BCR-ABL1-associated genomic instability [8]. Distinct TKI frontline strategy pathways may be chosen to obtain long-term treatment end-points in the personalized treatment of de novo CML. Patient age, CML risk (based on Sokal, Euro/Hasford, EUTOS, and ELTS scoring systems), comorbidities, and the long-term aim of the TKI treatment (mainly prevention of disease progression with life-long TKI drug administration or treatment-free remission) are the main cornerstones for choosing the frontline TKI strategy in CML [2,9].

**Pathway 1 (Imatinib as the Frontline TKI for CML):** Treatment with oral generic imatinib mesylate at 400 mg daily can be prescribed for any patient with CML as the initial therapy. Switching to a second-generation TKI may be considered in the case of resistance or intolerance during the CML follow-up period. The rational reasons for choosing this path are pharmacoeconomy, better tolerability, and less toxicity of imatinib with regard to second-generation TKIs. Furthermore, there is no difference of frontline dasatinib/nilotinib/bosutinib compared to imatinib in terms of survival [2].

**Pathway 2 (Second-Generation TKI as the Frontline Drug for CML):** Second-generation TKIs (nilotinib, dasatinib, bosutinib) may be administered to patients at high Sokal disease risk of CML for the prevention of disease progression and blastic crisis. The determination of disease risk may be defined using the Sokal, Euro/Hasford, EUTOS, or ELTS scoring systems [10]. The rationale for this path is the prevention of disease progression, accelerated disease, and blastic crisis in high-risk patients. CML patients with higher percentages of blasts, basophils, and eosinophils and those with thrombocytosis, BM fibrosis, and massive splenomegaly are candidates for frontline second-generation TKI therapy [11]. Relatively young CML patients representing the target subpopulation for treatment-free remission (TFR) should also be selected for the frontline nilotinib or dasatinib approach. However, there is no overall survival advantage between frontline imatinib and second-generation TKI approaches [12,13,14]. Therefore, imatinib 400 mg treatment shall be chosen for patients with drug/disease-associated comorbidities, for whom TFR is not a target [2].

In clinical practice, any TKI (imatinib, nilotinib, bosutinib, or dasatinib) as frontline therapy can be chosen with the optimization of the dosage with regard to the individual disease/patient characteristics, life expectancy, lifestyle, and comorbidities. TKI dosages (for example, imatinib 300 vs. 400 vs. 600 mg; dasatinib 50 vs. 100 vs. 140 mg; nilotinib 600 vs. 800 mg; ponatinib 15 mg vs. 30 mg; bosutinib 300 mg vs. 500 mg) could be tailored based on the tolerability, side effects, and BCR-ABL1 levels of the CML patients. The doses of TKIs shall be adopted based on the phase of CML and the line of TKI therapy. Lower starting TKI doses for the sake of tolerability should be titrated up to the standard doses in order to get hematological, cytogenetic, and molecular responses with the observation of toxicity, compliance, and tolerability. The rationale for the TFR path, i.e. frontline second-generation TKIs, is to obtain faster and deeper molecular responses including MR4.5 for TKI drug cessation [14,15]. The EURO-SKI trial was performed with molecular responders of MR4 with TKI-free long-term remissions, representing an advantage of survival without TKI toxicities, which may be referred to as “functional cure” [16]. Although the most significant literature experience with TKI discontinuation is with imatinib, patients with two-year administration of second-generation TKIs and a two-year duration of MR4.5 are ideal candidates for TKI drug discontinuation [2,16,17].

The response to TKI drug treatment in a patient with CML must be monitored to check for full hematological (CHR), complete cytogenetic (CCyR), and major molecular (MMR) remissions regardless of the path that has been chosen. The clinicobiological signs of normal hematopoiesis replacing Ph*(+) myeloid neoplasia should be investigated. Next-generation molecular analyses [18] may be incorporated in the follow-up of CML patients to search for genomic stability of the disease. Current disease guidelines such as those of the ELN or NCCN require CHR within the first month, CCyR within the first year, and MMR within 18 months of TKI therapy. BCR-ABL1 of less than 10% within the first 3 months after TKI is a very good prognostic sign called early molecular response (EMR). However, there is little evidence that switching to a second-generation TKI in the absence of EMR might produce better disease outcomes and prevention of disease progression [19,20]. Preliminary results of the DASCERN study implied that CML patients without EMR to imatinib at 3 months who switched to dasatinib had a significantly increased rate of MMR at 12 months when compared to patients receiving imatinib mesylate. Longer follow-up duration is certainly required to assess the impact of early switching of dasatinib at 3 months on the overall survival of patients [21].

Long-term adverse events associated with the chronic usage of TKI drugs described by the ELN [22] represent an important emerging challenge in everyday clinical practice for CML. Side effects of TKIs are generally mild to moderate and easy to manage in the middle periods of CML therapy [22]. Provisional discontinuation of the drug may be a choice in the case of serious adverse events. Close attention should be paid to drug-drug interactions [23]. Cardiovascular toxicity with ponatinib and nilotinib, pulmonary toxicity with dasatinib, and gastrointestinal/metabolic toxicities with bosutinib and nilotinib may require specific follow-up strategies for early adverse event detection and proper clinical management [24]. If properly managed, TKI therapies are well tolerated with improvement of the drug-related symptoms in due course with a few dose reductions or short drug holidays [25].

## Salvage Strategies in CML Patients

Salvage strategies in CML mainly depend on the alternative unused TKIs and allografting if all of the TKIs were used with a T315I mutation. Decision-making in multi-TKI-resistant CML should rely on the type of first-line treatment, type of resistance (TKI mutation, TKI failure, TKI intolerance, TKI incompliance), phase of disease, and transplant risk score of the patient. Before the consideration of TKI alteration during life-time management of CML, drug dose adjustments, such as TKI dose decrements in the event of adverse events and increments in the presence of insufficient BCR-ABL1 control, shall be performed. The optimal salvage therapeutic strategy for CML will avoid both over- and under-treatment. CML over-treatment may be described as aggressive clinical intervention. For instance, the early/inappropriate decision to apply a very risky hematopoietic stem cell transplantation (HSCT) in a CML patient receiving a given second-generation TKI and exhibiting inadequate response, in which a third-generation TKI or dose increments would produce a better outcome, requires careful consideration. On the other hand, the inability to detect warning signs of relapse/resistance in follow-up resulting in TKI failure and/or blastic crisis may also be considered as inappropriate management. ABL mutations of T315I, Y253H, E255K, E255V, F359V, F359C, and F359I are poorly sensitive to nilotinib and T315I, T315A, F317L, F317V, F317I, F317C, and V299L are the mutations poorly sensitive to dasatinib. Ponatinib is the only TKI for T315I before HSCT. The most challenging situations in patients with CML are resistance to all available TKIs in patients who inability to undergo transplantation, or recurrence after HSCT, especially into blastic crisis [26]. The fourth-generation drug asciminib, a specific TKI targeting the BCR-ABL1 myristoyl-binding site, an allosteric regulatory domain, and PF-114 mesylate [27], have the potential to treat patients with resistance to ATP-binding-site TKIs, including T315I [28,29]. CML leukemic stem cells expressing IL-1RAP can be targeted by CAR-T cells (chimeric antigen receptor-engineered T lymphocytes) [30]. Manipulations of CML stem cells [31], neoplastic bone marrow niche trafficking control [32], and the CRISPR/Cas9 system with nanocarriers [33] seem to be future research areas in the field of CML therapy.
